# Neuroprotection Against Parkinson’s Disease Through the Activation of Akt/GSK3β Signaling Pathway by Tovophyllin A

**DOI:** 10.3389/fnins.2020.00723

**Published:** 2020-07-09

**Authors:** Yanjun Huang, Lirong Sun, Shuzhen Zhu, Liu Xu, Shuhu Liu, Chunhua Yuan, Yanwu Guo, Xuemin Wang

**Affiliations:** ^1^Key Laboratory of Mental Health of the Ministry of Education, Guangdong-Hong Kong-Macao Greater Bay Area Center for Brain Science and Brain-Inspired Intelligence, Guangdong Province Key Laboratory of Psychiatric Disorders, Department of Neurobiology, School of Basic Medical Sciences, Southern Medical University, Guangzhou, China; ^2^Department of Neurology, Zhujiang Hospital, Southern Medical University, Guangzhou, China; ^3^Department of Neurosurgery, Zhujiang Hospital, Southern Medical University, Guangzhou, China

**Keywords:** Tovophyllin A, Parkinson’s disease, apoptosis, Akt, GSK3β

## Abstract

Parkinson’s disease (PD) is one of the most prevalent and life-threatening neurodegenerative disease and mainly characterized by lack of sufficient dopaminergic neurons in the substantia nigra pars compacta (SNc). Although current treatments help to alleviate clinical symptoms, effective therapies preventing neuronal loss remain scarce. Tovophyllin A (TA), one of the xanthones extracted from *Garcinia mangostana* L. (GM), has recently been reported to play a beneficial role in the therapy of neurodegenerative diseases. In our research, we explored whether TA has protective effects on dopaminergic neurons in PD models. We found that TA significantly reduced apoptotic cell death in primary cortical neurons treated with 1-methyl-4-phenyl pyridinium (MPP^+^) or paraquat (PQ) in the *in vitro* PD model. In an *in vivo* acute PD model induced by 1-methyl4-phenyl-1,2,3,5-tetrahydropyridine (MPTP) treatment, TA also attenuated the resulting behavioral dysfunctions and dopaminergic neuron loss. In the collected brain tissues, TA increased the phosphorylation of Akt and GSK-3β, which may be related to TA-mediated dopaminergic neuronal protective effects. In summary, our results illustrated that TA is a powerful cytoprotective agent for dopaminergic neurons in the MPTP-induced PD model, suggesting TA as a possible therapeutic candidate for PD.

## Introduction

Parkinson’s disease (PD), a long-term and complex movement disorder, is hallmarked by the classical motor features, including slowness of movement, resting tremors, stiffness, and postural instability (Dauer and Przedborski, 2003; Dexter and Jenner, 2013; Kalia and Lang, 2015). In recent years, non-motor symptoms have also been highly concerned, such as olfactory impairments, sleep disorders, autonomic dysfunctions, and emotional disturbances (Kalia and Lang, 2015). The major pathology of PD is characterized by the gradual and massive loss of dopaminergic neurons in the substantial nigra pars compacta (SNc), resulting in a reduction in dopamine (DA) levels (Martinez et al., 2017). Although the precise pathogenic mechanism of the neurodegeneration in PD is not yet fully understood, various factors that alone or together, such as oxidative stress (OS), neuroinflammation and mitochondrial toxins, have been implicated to the progressive impairments of dopaminergic neurons (Moon and Paek, 2015; Morris and Berk, 2015; Hu et al., 2019). It is widely accepted that energy deficits caused by mitochondrial dysregulation and oxidative stress induced by high levels of unstable radicals could mightily contributed to neurodegenerative diseases (Iarkov et al., 2020).

1-methyl4-phenyl-1,2,3,5-tetrahydropyridine (MPTP) and 1-methyl-4-phenyl pyridinium (MPP^+^) are well-known neurotoxins. MPP^+^, the active metabolite of MPTP, is transported into dopaminergic neurons by the dopamine transporter (DAT). It is then isolated into synaptosomal vesicles or enriched in the mitochondria, where it promotes the production of free radicals (Jackson-Lewis and Przedborski, 2007; Motyl et al., 2018). As dopaminergic neurons are highly sensitive to these compounds, MPP^+^ and MPTP are widely used to establish diverse PD models, both *in vitro* and *in vivo* (Cui et al., 2013; Jantas et al., 2014; Zhao et al., 2016). When response to the toxicology of MPP^+^ and MPTP, necrotic and apoptotic mechanisms of cell death occurred.

Current pharmacological therapeutics such as dopamine precursor, L-DOPA and DA receptor agonists could ameliorate clinical symptoms, and the classical surgical treatment called deep brain stimulation (DBS) can also improve the symptoms, however, all these approaches rarely alleviate dopaminergic neuronal loss (Rizek et al., 2016; Iarkov et al., 2020). Thus, identifying new neuroprotectants that reduce neuronal loss is of great significance for the treatment of PD.

Glycogen synthase kinase-3β (GSK-3β) is tightly related to the loss of dopaminergic neurons in PD models and MPP^+^-caused neuronal death (Golpich et al., 2015; Chen et al., 2017; Yue et al., 2017). It can be inactivated by Akt and other kinases by phosphorylating of the single serine residue (Ser9), which is located in the regulatory N-terminal domain (Frame and Cohen, 2001; Beaulieu, 2007). Akt is a key player in the phosphoinositide 3-kinase (PI3K)/protein kinase B (Akt/PKB) signaling pathway which is essential for protecting neurons from oxidative stress (Lu et al., 2011). Activation of this pathway is considered to improve cell survival and protect from the apoptosis (Dudek et al., 1997). As a result, the cascade of PI3K/Akt/GSK-3β is considered to serve a critical role in the pathogenesis of PD.

*Garcinia mangostana* L. (GM, Guttiferae family), also recognized as mangosteen, is native to the Southeast Asia countries. Seeds and pericarps of this tropical fruit have been used for a long time in traditional medicinal actions in these regions (Ovalle-Magallanes et al., 2017). The major phytoconstituent contents in the species are isoprenylated xanthones, a group of heterocyclic metabolites with a xanthene-9-one framework. Xanthones have a number of biological effects including anti-oxidation (Pedraza-Chaverri et al., 2008), anti-tumor (Hafeez et al., 2014), anti-nociception (Fidanboylu et al., 2011), anti-inflammation (Jang et al., 2012; Wang et al., 2016; Tousian Shandiz et al., 2017), neuroprotection (Wang et al., 2012), and anti-obesity (Liu et al., 2015). Tovophyllin A (TA), one of the xanthones mainly extracted from the mangosteen pericarp, has been displayed to protect mitochondrial functions and against oxidative stress (Ibrahim et al., 2018).

However, the neuroprotection of TA and its potential mechanisms in PD models remain to be further explored. In this research, we showed the neuroprotection of TA against MPP^+^/PQ-induced cytotoxicity in primary neurons and investigated its potential therapeutic effect in a mouse PD model. The results indicated that TA modulated the pathway of Akt/GSK-3β, which may contribute to TA-induced dopaminergic neuron protection.

## Materials and Methods

### General Experimental Procedures

NMR and HRESIMS spectra were recorded by a Bruker ADVANCE-600 (600 MHZ) Instrument (Bruker Biospin, Zurich, Switzerland) and UPLC-QTOF-MS (Waters Ltd., Milford, MA, United States) in positive ion mode, respectively. Silica gel (80–100 and 200–300 mesh) obtained from Qingdao Haiyang Chemical Co., Ltd., Qingdao, China, and Sephadex LH-20 was purchased from Pharmacia Fine Chemical Co., Ltd., Uppsala, Sweden. The HSGF_254_ (Yantai Jiangyou Silica Gel Co., Ltd., Yantai, China) was used for thin-layer chromatography (TLC). Spots were visualized by spraying with 10% sulphuric acid in ethanol (*v*/*v*) followed by heating the silica gel plates. All reagents used were analytical-grade and purchased from the Tianjin Fuyu Fine Chemical Industry Co., Ltd. (Tianjin, China).

### Plant Material

Fresh *G. mangostana* L. from Thailand were obtained from Guangzhou fruit market in January 2017. A dry voucher specimen (#20170316GM) has been deposited in the herbarium of the School of Basic Medical Science, Southern Medical University, China.

### Extraction and Isolation of TA

**FIGURE 1:**
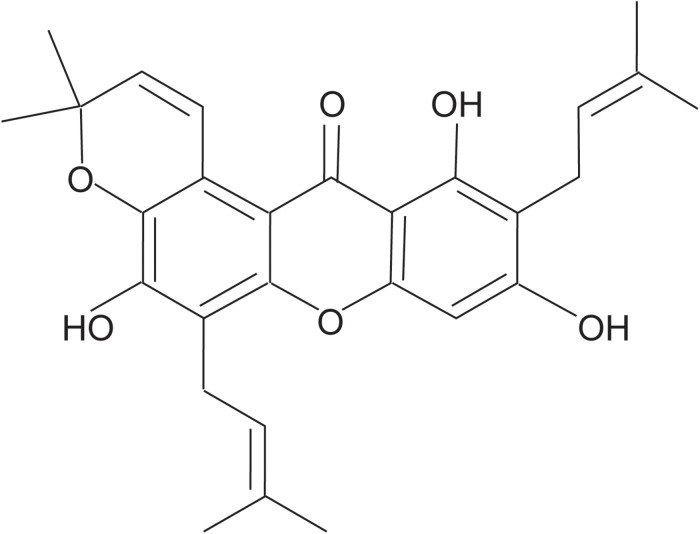
Chemical structure of Tovophyllin A.

Tovophyllin A (Figure 1) was extracted and purified from the pericarp of *G. mangostana* L. In brief, air-dried fruit pericarp (1 kg) was ground and extracted with 95% ethanol (10 L × 3) at room temperature for 24 h. The mixture was evaporated under a vacuum to produce a crude extract (188 g). The crude extract was fractionated between methylene chloride and water to produce a methylene chloride fraction (5.4 g). The methylene chloride soluble fraction was dissolved in methylene chloride/ethyl acetate (3:1) and applied to a silica gel column (5 *×* 920 mm, 200–300 mesh). The sample was eluted with petroleum ether (1 L), dichloromethane (1 L), and dichloromethane: methanol (100:1; 2 L) to obtain impure TA. It was then purified using RP_18_ column chromatography eluted with a MeOH : H_2_O gradient to give TA (18 mg) as a yellow powder. TA purity was shown to be >98% based on HPLC.

### Spectral Data of TA

Tovophyllin A: yellow powder; C_28_H_30_O_6_; ^1^H NMR (CDCl_3_, 600 MHz): δ_*H*_ 6.35 (s, H-4), 13.77 (s, 1-OH), 3.47 (d, J = 7.2 Hz, H-1′), 5.27 (tq, J = 7.2, 1.2 Hz, H-2′), 1.85 (s, H-4′), 1.87 (s, H-5′), 3.57 (d, J = 7.2 Hz, H-1″), 5.29 (tq, J = 7.2, 1.2 Hz, H-2″), 1.68 (s, H-4″), 1.78 (s, H-5″), 8.00 (d, J = 10.2 Hz, H-1′′′), 5.78 (d, J = 10.2 Hz, H-2′′′), 1.49 (s, H-4′′′), 1.49 (s, H-5′′′); ^13^C NMR (CDCl_3_,150 MHz): δ_*C*_ 160.4 (C-1), 108.2 (C-2), 161.6 (C-3), 93.4 (C-4), 155.3 (C-4a), 151.0 (C-4b), 115.2 (C-5), 148.6 (C-6), 135.9 (C-7),117.2 (C-8), 108.4 (C-8a), 103.7 (C-8b), 182.9 (C-9), 21.4 (C-1′), 121.0 (C-2′), 136.5 (C-3′), 25.8 (C-4′), 17.9 (C-5′), 22.6 (C-1″), 121.1 (C-2″), 132.6 (C-3″), 25.9 (C-4″), 17.9 (C-5″), 121.5 (C-1′′′), 131.3 (C-2′′′), 76.8 (C-3′′′), 27.4 (C-4′′′), 27.4 (C-5′′′); HRESIMS *m/z* 485.1940 (M + Na)^+^ (C_28_H_30_O_6_Na, calcd for 485.1935), HRESIMS *m/z* 461.1975 (M−H)^–^ (C_28_H_29_O_6_, calcd for 461.1970). The structure of this compound was assigned by NMR and validated by comparing with the literature and spectroscopic and physical data (Ibrahim et al., 2018).

### Chemicals and Antibodies

1-methyl4-phenyl-1,2,3,5-tetrahydropyridine (MPTP), 1-methyl-4-phenyl pyridium (MPP^+^), paraquat (PQ), and 3-(4,5-Dimethylthiazol-2-yl)-2,5-diphenyltetrazolium bromide (MTT) were obtained from Sigma-Aldrich (St. Louis, MO, United States). Anti-tyrosine hydroxylase (TH) antibody (#ab112) was obtained from Abcam (Cambridge, United Kingdom). Antibodies against phospho-Ser473-Akt (#9271S), total Akt (#9272S), phospho-Ser9-GSK3β (#9336S), total GSK3β (#9315S) were purchased from Cell Signaling Technology (Danvers, MA, United States). Anti-GAPDH (#60004-1-Ig) was purchased from ProteinTech Group (Rosemont, IL, United States). Secondary antibodies conjugated to Alexa 488 were purchased from Invitrogen (Carlsbad, CA, United States).

### Cultures of Primary Cortical Neurons

As previously described (Qin et al., 2015), primary cortical neurons were obtained from D0 C57BL/6 mice and cultured in neurobasal medium supplemented with 2% B27 and 25 μM glutamate on poly-L-lysine-coated plates in a 5% CO_2_ incubator at 37°C. Every 3 days, half of the culture medium was changed with fresh medium without glutamate.

### Analysis of Cell Viability by MTT Assay

Cell viability was evaluated by the colorimetric MTT assay. Briefly, primary neurons were treated with different concentrations of TA, and then exposed to 100 μM MPP^+^ or PQ. After 24 h, MTT was added directly to the cells, which were incubated at 37°C for another 4 h. After removing the medium, 500 μl of DMSO was added to each well. Hundred microliters of the supernatant was transferred into a 96-well plate which were shaken on a microplate shaker to make sure that the MTT formazan crystals were completely dissolved. The absorbance was measured at 570 nm by a spectrophotometer. All values were normalized by subtracting the blank value measured with only DMSO. The absorbance value of the experimental groups was expressed as a percentage of the control group which was set as 100% viability.

### Experimental Animals and MPTP Administration

Male C57BL/6 mice were obtained from the Animal Center of Southern Medical University (Guangzhou, Guangdong Province, China). The animals (8–10 weeks old, 25–28 g) were raised in SPF conditions (temperature: 22 ± 1°C) under a 12/12 h light-dark cycle with lights on 8:00 a.m. Food and water were available *ad libitum*. MPTP solution in saline was freshly prepared and injected intraperitoneally (i.p.) in four separate doses in 2 h intervals within a single day, in a total dose of 40 mg/kg of body weight (bw). Behavioral tests were performed 7 days after the treatment, then the mice were sacrificed for the collection of the brain tissues. All experimental procedures in this research were approved by the animal ethical committee of Southern Medical University.

### Open Field Test

The open field test was performed by using a rectangular chamber (40 cm × 40 cm × 30 cm) which is made of polyvinyl chloride (PVC). Each mouse was put in the peripheral zone and allowed to explore the open field arena freely for 5 min. The behavioral responses were recorded using software EthoVision 7.0 (Noldus). At the end of each trial, 70% ethanol was used to clean the open field arena before placing the next animal. Data was recorded and analyzed in a blinded fashion.

### Rotarod Test

Before MPTP treatment, the mice were trained on the rotarod three times with 20-min intervals between trials every day for three consecutive days (nine trials in total). Each mouse was trained by gradually increasing the speed of rotation on each day: 5–10 rpm (accelerated at 1 rpm/5 s) on day 1, 11–15 rpm (accelerated at 1 rpm/5 s) on day 2, and 16–20 rpm (accelerated at 1 rpm/5 s) on day 3. The rod was rotated manually (nonautomated). Seven days after the MPTP injections, the mice were re-evaluated with a constant acceleration from 4 to 40 rpm over 300 s. The average latency to fall from the rod of each mouse was measured and calculated.

### Immunofluorescence Staining

Mice were perfused transcardially with 4% paraformaldehyde (PFA) in phosphate buffer (pH 7.4) and the brains were post-fixed overnight at 4°C. After the cryoprotection in 30% sucrose solution over 3 days, coronal slices at a thickness of 40 μm were obtained from the frozen tissues using a sliding blade microtome and then collected on microscope slides. Immunofluorescence staining method was performed according to the previously reported (Luo et al., 2018). The SNc region of brain sections was permeabilized in PBS with 0.3% Triton X-100 for 30 min and blocked in PBS with 5% normal goat serum for 2 h at room temperature. Sections were incubated with specific primary antibodies (1:200 dilution for anti-TH) at 4°C overnight. After washing with PBS, slides were incubated in the solution containing Alexa Fluor 488-conjugated secondary antibody for 2 h at room temperature. The nuclei were stained with 4′,6-diamidino-2-phenylindole (DAPI) and the brain tissues were imaged under confocal microscopy.

### Quantitative Analysis of TH-Positive Cells in the SNc

To investigate the loss of neurons in the SNc, serial section analysis of the total number of TH-positive neurons were shown by immunofluorescence staining. Four to six mice were used per group. The number of TH-positive neurons with obviously visible processes and nuclei was calculated on four sections for each subject by an investigator blind to group allocation. The serial sections were cut at 40 μm.

### Western Blot

The protein concentration of each sample was determined using BCA Protein Assay Kit (Pierce). Samples (20–40 μg) were separated by 10% SDS-polyacrylamide gels and transferred to polyvinylidene difluoride (PVDF) membranes. The protein levels were determined using the following primary antibodies: anti-TH (1:200), phospho-Ser473-Akt (1:1000), total Akt (1:1000), phospho-Ser9-GSK3β (1:1000), total GSK3β (1:1000), and anti-GAPDH (1:10,000), overnight at 4°C, followed by 1 h incubation with the proper horseradish peroxidase-conjugated secondary antibodies (1:4000). Proteins were visualized using the enhanced chemiluminescence (ECL) reagent and densitometric analysis was performed using the ImageJ software. Densitometric analysis is displayed as relative optical density.

### Statistical Analyses

All values are presented as mean ± SEM. Statistical analysis was performed using one-way ANOVAs followed by *post hoc* Bonferroni test for multiple comparisons. *p* < 0.05 or *p* < 0.01 was considered statistically significant. Each experiment was performed at least three times.

## Results

### TA Significantly Protected Primary Cultured Cortical Neurons Against MPP^+^-Induced or PQ-Induced Neurotoxicity

**FIGURE 2:**
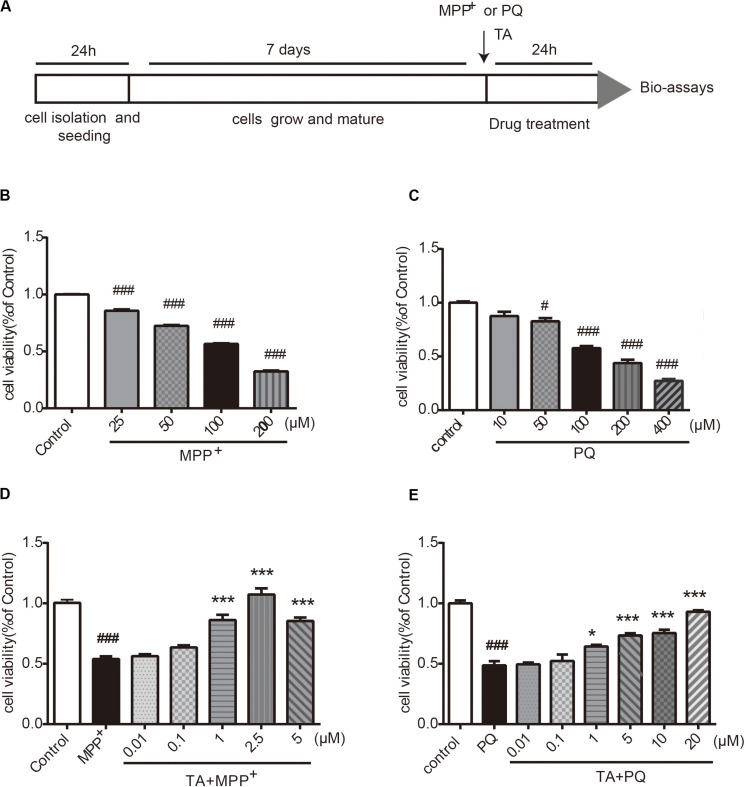
The effects of TA on MPP^+^ and PQ-induced cell death in primary cultured cortical neurons. **(A)** Timeline for the cell viability assay. **(B,C)** Dose-dependent decrease in cell viability with different doses of MPP^+^/PQ or saline in cortical neurons. **(D,E)** TA protected neurons against MPP^+^/PQ-induced neurotoxicity. Data are expressed as percentage of control (*n* = 6). One-way ANOVA followed by *post hoc* Bonferroni test: ^#^*p* < 0.05, ^###^*p* < 0.001 compared with the control group; *p < 0.05, ***p < 0.001 compared with the MPP^+^ group or the PQ group. All values are expressed as mean ± SEM.

The timeline for the evaluation of TA was shown in Figure 2A. First, we examined the neurotoxicity of MPP^+^ and PQ in primary cultured neurons. As depicted in Figures 2B,C, MPP^+^ and PQ caused significant reductions of cell viability in a dose-dependent manner compared with control group. As a result, 100 μM MPP^+^ or PQ was used for subsequent experiments.

The protective effects of TA on MPP^+^-toxicity and PQ-toxicity in primary cultured neurons were investigated by MTT assay. Briefly, cells were co-treated with TA of different concentrations and 100 μM MPP^+^ or PQ for 24 h. As shown in Figure 2D, the MPP^+^-treated group showed significantly decreased cell viability compared to control group. Interestingly, TA treatment protected neurons from cell death caused by MPP^+^, especially at the concentrations of 1 and 2.5 μM. Similarly, as shown in the Figure 2E, TA also exerted protective effects in PQ-treated cells. The results suggest that TA protects primary cortical neurons from MPP^+^ and PQ cytotoxicity.

### TA Ameliorated MPTP-Induced Behavioral Deficits

**FIGURE 3:**
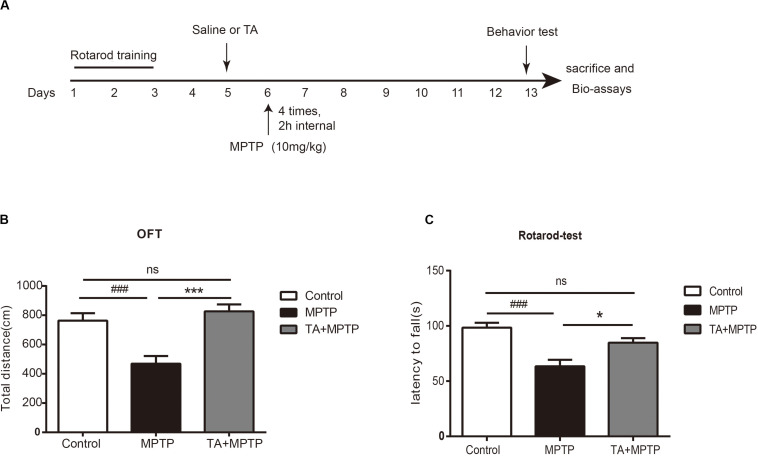
TA ameliorated MPTP-induced behavioral deficits. **(A)** Timeline of experiments *in vivo*. **(B)** Total distance traveled by different cohorts, namely control, MPTP and TA+MPTP, in the open field test. **(C)** The latency to fall of various cohorts assessed by rotarod test. One-way ANOVA followed by *post hoc* Bonferroni test: ^###^*p* < 0.001 compared with the control group; *p < 0.05, ***p < 0.001 compared with the MPTP group (*n* ≥ 8). Data are shown as mean ± SEM.

To evaluate the TA-mediated protective effect against MPTP-induced neurotoxicity in the *in vivo* model, as outlined in Figure 3A, we first detected whether TA could improve the motor dysfunctions in MPTP-treated mice by open field and rotarod tests. As shown in Figure 3B, the total distance in the open field test traveled by MPTP-treated cohort 7 days post-injection was remarkably reduced compared with that of the saline group (MPTP vs control, *p* < 0.001), validating MPTP-induced locomotive impairments. These impairments were reduced when pre-treated with TA (5 mg/kg) as the distance increased obviously (TA + MPTP vs MPTP, *p* < 0.001) and had no significance compared to the control cohort (TA + MPTP vs control, *p* > 0.05).

In the rotarod test, as shown in Figure 3C, the latency to falling of the rotarod for the MPTP cohort decreased dramatically compared with that of control cohort 7 days post-administration of MPTP (MPTP vs control, *p* < 0.001), affirming the motor impairments in MPTP-treated mice. Compared with the MPTP-treated cohort, the latency to fall in the TA pre-treatment cohort increased remarkably (TA + MPTP vs MPTP, *p* < 0.05). In addition, the latency to fall in the TA pre-treatment cohort was comparable with that of the control cohort (TA + MPTP vs control, *p* > 0.05). Thus, pre-treatment of TA has shown to rescue motor deficits resulted from MPTP toxicity *in vivo*.

### TA Alleviated MPTP-Induced Dopaminergic Neuronal Loss *in vivo*

**FIGURE 4:**
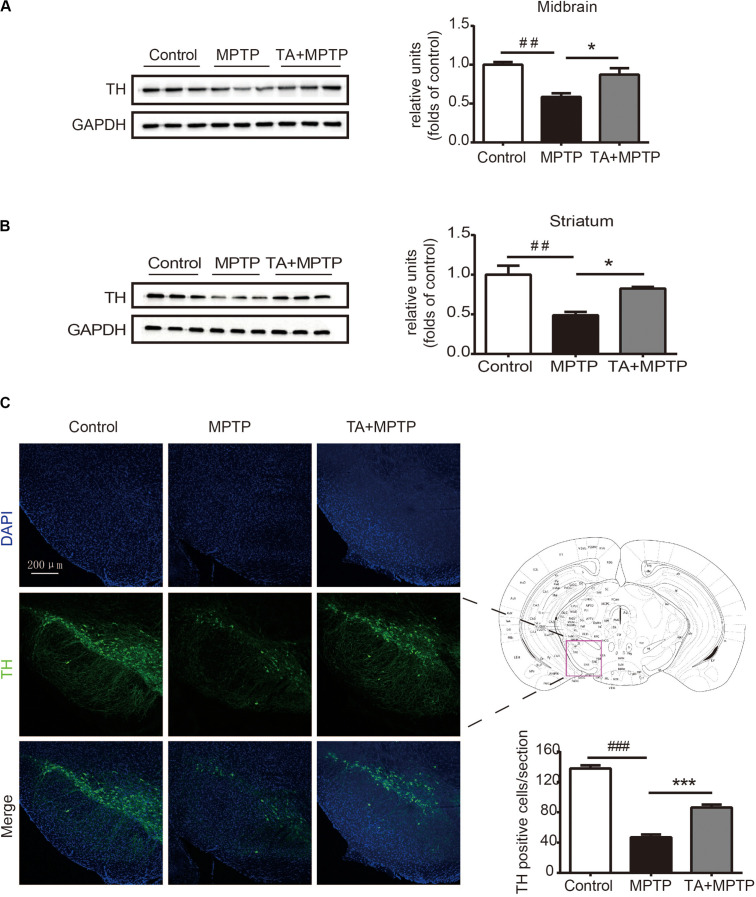
TA treatment attenuated MPTP-induced decrease in tyrosine hydroxylase (TH) expression in mice. **(A,B)** Western blot analysis of the level of TH in the mouse midbrain and striatum. The relative optical density of TH was normalized by the internal loading control GAPDH. **(C)** Immunofluorescence staining with anti-TH antibody in the substantia nigra pars compacta (SNc). Scale bar: 200 μm. One-way ANOVA followed by *post hoc* Bonferroni test: ^##^*p* < 0.01, ^###^*p* < 0.001 compared with the control group; *p < 0.05, ***p < 0.001 compared with the MPTP group (*n* ≥ 3). Data are shown as mean ± SEM.

Next, we investigated the survival of dopaminergic neurons in mice after MPTP administration. Tyrosine hydroxylase (TH), as a particular biomarker for midbrain dopaminergic neurons, was detected by western blot analysis and immunofluorescence staining in the substantia nigra pars compacta (SNc). As shown in Figures 4A,B, the level of TH dramatically decreased in the midbrain tissues and striatum 7 days after MPTP treatment, while TA obviously alleviated the TH reduction in MPTP-treated mice. In Figure 4C, TH in the SNc was detected by immunofluorescence staining to visualize the number of dopaminergic neurons. MPTP caused the number of dopaminergic neurons decrease in SNc, whereas TA powerfully improved the survival of these neurons in MPTP-treated mice. Our results illustrated that TA treatment can effectively preserve the expression of TH in midbrain and dramatically improve the survival of dopaminergic neurons against neurotoxicity induced by MPTP.

### TA Activated the Akt Pro-survival Pathway in MPTP Treated Mice

**FIGURE 5:**
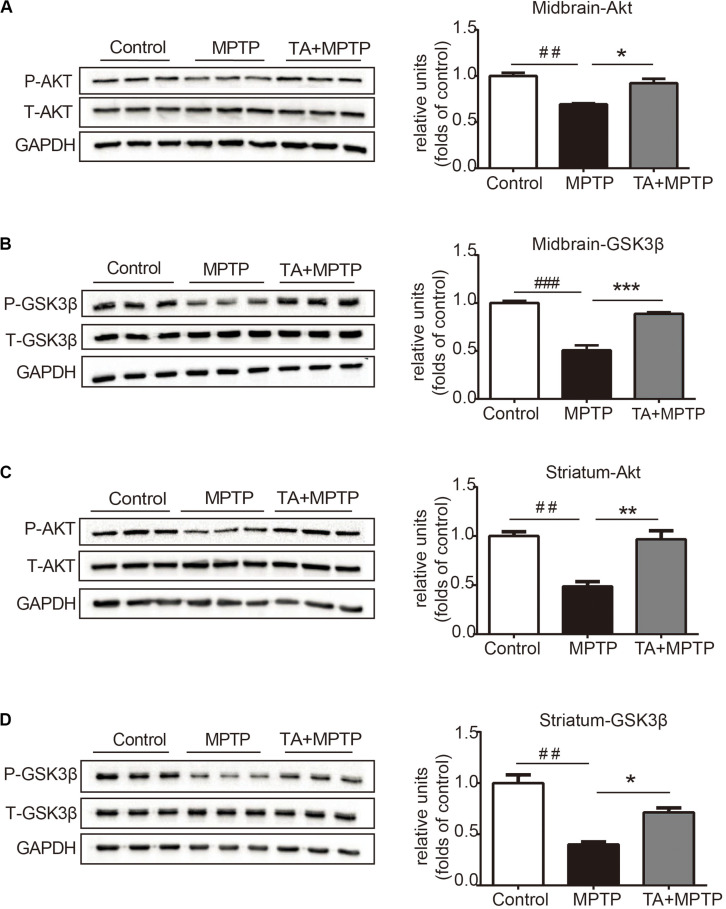
TA activated Akt and attenuated MPTP-induced GSK-3β activation. **(A,B)** Protein levels of p-Akt, Akt, p-GSK3β, GSK3β, and GAPDH were determined by western blot analysis in the midbrain. **(C,D)** Protein levels of p-Akt, Akt, p-GSK3β, GSK3β, and GAPDH were determined by western blot analysis in the striatum. One-way ANOVA followed by *post hoc* Bonferroni test: ^##^*p* < 0.01, ^###^*p* < 0.001 compared with the control group; *p < 0.05, **p < 0.01, ***p < 0.001 compared with the MPTP group (*n* ≥ 3). The data are presented as mean ± SEM.

The PI3K / Akt/PKB signaling pathway is of great importance in cell survival, proliferation and differentiation (Babichev et al., 2016; Zhang et al., 2016). Akt phosphorylate alters the activity of its downstream substrate GSK-3β, which has been shown to result in DA neuronal reduction caused by MPTP and other insults. Thus, we evaluated whether TA promotes neuron survival by regulating of the Akt/GSK-3β signaling pathway. As shown in Figure 5A, MPTP administration decreased the phosphorylation level of Akt in the midbrain, while having no effect on total Akt. TA treatment, however, alleviated the inhibitory effect on the phosphorylation of Akt at Ser473 caused by MPTP and reversed the pAkt/Akt ratio significantly (Figure 5A). Similarly, MPTP administration induced activation of GSK3β, and TA treatment abolished the MPTP-induced activation of GSK-3β at Ser9 (Figure 5B). Next, we further investigated the changes in the Akt/GSK3β pathway in the striatum. As shown in Figures 5C,D, results found were consistent with western blot data. These findings indicate that the DA neuroprotective effects of TA may through the regulation of the Akt/GSK-3β pathway.

## Discussion

Parkinson’s disease is a frequent type neurodegenerative disease with uncertain precise underlying mechanisms and no clinically effective treatment available (Li et al., 2019). Since the progressive loss of dopaminergic neurons in the SNc is the most signature pathological features, identifying effective neuroprotectants is an important direction for the research in novel therapies (Iarkov et al., 2020). In this study, our present data demonstrated (1) TA significantly increased cell viability in neurotoxin (MPP^+^ or PQ)-injured primary cortical neurons, consistent with previous findings by Jaisin et al. (2018); (2) MPTP-induced behavioral impairments and DA neuronal loss were significantly attenuated by co-treatment with TA *in vivo*; (3) TA administration activated Akt and increased GSK-3β phosphorylation level in the striatum and midbrain of MPTP-treated mice. These data provide clear evidence that TA is an effective neuroprotectant against MPP^+^/MPTP lesions on DA neurons, which may be through the regulation of the Akt/GSK-3β signal pathway.

*Garcinia mangostana* L. has abundant pool of xanthones that present a variety of pharmacological activities (Ibrahim et al., 2018). The diversity of actions existed in mangosteen xanthones indicate that lots of signaling pathways involved in different pathologies have been targeted by these compounds, and allows them to become precious sources for developing new medicines for the treatment of chronic and degenerative diseases (Ovalle-Magallanes et al., 2017). For instance, α-mangostin, a major compound of the GM pericarps, was shown to prevent the fibril formation and dissociate Aβ aggregation, which is beneficial to attenuating Aβ oligomers-induced neurotoxicity in Alzheimer’s disease (Wang et al., 2012). Moreover, α-mangostin can also alleviate the aggregation of α-synuclein and loss of TH in rotenone-treated SH-SY5Y cells, suggesting potential neuroprotective effects of α-mangostin against PD-related neuronal injury (Hao et al., 2017). In addition, similar to antioxidants, xanthones protect cells from lead-induced damages in the kidney by decreasing oxidative stress, downregulating inflammation factors and inhibiting apoptosis (Rana et al., 2020). TA, which is specially derived from GM pericarps, has recently been reported to serve a protective effect in acetaminophen-induced hepatic damage by activating Nrf2 and down regulating NF-κB pathways (Ibrahim et al., 2018). The etiopathogenesis of aging-related neurodegenerative disease is correlated with various processes, including neuroinflammation, oxidative stress, and abnormal dopaminergic system function (Motyl et al., 2018). Overproduction of reactive oxygen species (ROS) during oxidative stress and the disturbance of antioxidant defense have been considered as crucial causative factors in PD (Guo et al., 2018). In the study, we investigated whether TA had neuroprotective effects on PD models both *in vitro* and *in vivo*. We found that TA directly protected primary cultured neurons against MPP+ and PQ insults (Figure 2). Moreover, TA administration remarkably alleviated MPTP-induced behavioral dysfunctions and DA neuron loss (Figures 3, 4), suggesting that TA may be a promising therapeutic approach in PD.

The PI3K/Akt cascade is a pro-survival pathway of great significance for the development of the nervous system. Akt is mainly activated by PI3K, and this pathway promotes cell survival and cytoprotection by phosphorylating various enzymes, including antioxidant proteins and pro-apoptotic regulators, as well as some transcription factors (Nakano et al., 2017; Yue et al., 2017). Inhibition of this cascade is correlated with neurodegeneration, particularly in PD (Hu et al., 2018). GSK-3 is involved in an array of progresses, such as metabolism, gene expression, proliferation and cell survival. Activities of the two isomers, GSK-3α and GSK-3β, are dependent on phosphorylation at specific sites (Golpich et al., 2015). GSK-3β, phosphorylated at Ser9, which is considered to be tightly associated with the pathogenesis of PD, has been proven to play a crucial role in neuronal apoptosis both in PD mouse model and postmortem brains of PD patients (Duka et al., 2009; Nagao and Hayashi, 2009). Akt inhibits GSK-3β function via phosphorylation at serine residue (Ser9 of GSK-3β) and thereby reduces apoptosis (Golpich et al., 2015; Norwitz et al., 2019). In our study, we found that MPTP decreased Akt activity and increased of GSK-3β function, consistent with previous studies (Hu et al., 2018). Remarkably, TA pre-treatment abolished MPTP-induced changes and increased p-Akt/Akt ratio while decreasing GSK-3β activity in mice. This likely contributed to TA-mediated DA neuronal protection and the resulting improvement in behavioral performances. In order to meet the higher level of energy consumption, the brain is enriched abundant mitochondria. The central nervous system, especially dopaminergic neurons, is susceptible to oxidative damage (Guo et al., 2018). As an important xanthone, TA might also have other important pharmacological properties, such as antioxidative, anti-bacterial, anti-inflammatory abilities. Its neuroprotection role might be achieved by ameliorating the vulnerability to oxidative attack, activating the MAPK pathway and attenuating the expression of inflammatory genes, such as TNF-α, IL-6, and INF-γ(Tousian Shandiz et al., 2017). Our study provides important evidences on the ability of TA to modulate the Akt/GSK-3β pathway in the mouse PD model. However, the relationship between neuroprotection against PD and the activation of Akt/GSK-3β by TA is to be confirmed using Akt/GSK-3β inhibitors. Moreover, whether α-mangostin and TA could synergistically protect dopaminergic neurons against neurodegeneration in PD, and the exact mechanism underlying TA-mediated regulation of the Akt/GSK-3β signaling pathway must be further investigated.

## Conclusion

In summary, our study demonstrated that TA has significant neuroprotective effects both in MPP^+^/PQ-injured primary cortical neurons and MPTP-induced PD mouse model. Collectively, TA may be a potent pharmacological candidate for preventing dopaminergic neuronal death and neurodegeneration in PD.

## Data Availability Statement

All datasets generated for this study are included in the article/supplementary material.

## Ethics Statement

The animal study was reviewed and approved by the animal ethical committee of Southern Medical University.

## Author Contributions

XW designed the experiments. LS extracted and isolated TA. YH, SZ, and LX performed the cell viability assays, behavioral tests, and the biochemical analysis. SL, CY, and YG analyzed the data. YH and LS wrote the manuscript with help from XW. All authors read and approved the final manuscript.

## Conflict of Interest

The authors declare that the research was conducted in the absence of any commercial or financial relationships that could be construed as a potential conflict of interest. The reviewer WY declared a shared affiliation with one of the authors, SZ, to the handling editor.
